# Increased expression of system large amino acid transporter (LAT)-1 mRNA is associated with invasive potential and unfavorable prognosis of human clear cell renal cell carcinoma

**DOI:** 10.1186/1471-2407-13-509

**Published:** 2013-10-30

**Authors:** Hironori Betsunoh, Takehiko Fukuda, Naohiko Anzai, Daisaku Nishihara, Tomoya Mizuno, Hideo Yuki, Akinori Masuda, Yoshiyuki Yamaguchi, Hideyuki Abe, Masahiro Yashi, Yoshitatsu Fukabori, Ken-Ichiro Yoshida, Takao Kamai

**Affiliations:** 1 Department of Urology, Dokkyo Medical University, 880 Kitakobayashi, Mibu, Tochigi 321-0293, Japan; 2 Department of Pharmacology, Dokkyo Medical University, Mibu, Tochigi, Japan

## Abstract

**Background:**

The system L amino acid transporter (LAT) has an important role in the transport of various amino acids, and there have been reports about the relation of this system to cancer. Although LATs are highly expressed in the kidneys, little is known about their influence on human renal cancer.

**Methods:**

To clarify the role of LATs in human clear cell renal cell carcinoma (RCC), we investigated the expression of mRNAs for LAT1, LAT2, LAT3, LAT4, and 4F2hc in clear cell RCC tissues. The mRNAs of these five genes were analyzed by the real-time reverse transcription polymerase chain reaction in matched sets of tumor and non-tumor tissues obtained at operation from 82 Japanese patients with clear cell RCC. We also measured phosphorylated S6 ribosomal protein (Ser-235/236) proteins levels in 18 paired tumor and non-tumor tissues of the patients by Western blotting.

**Results:**

Expression of LAT1 mRNA was significantly increased in tumor tissue compared with non-tumor tissue, while expression of LAT2 and LAT3 mRNAs was reduced. There was no difference in the expression of LAT4 and 4F2hc mRNAs between tumor and non-tumor tissues. Increased expression of LAT1 mRNA was associated with less differentiated tumors, local invasion, microscopic vascular invasion, and metastasis. Kaplan-Meier survival analysis showed that a higher serum LAT1 mRNA level was associated with a shorter overall survival time. Phosphorylated S6 ribosomal protein levels were associated with metastatic potential. LAT1 mRNA levels positively correlated with phosphorylated S6 ribosomal protein proteins levels in primary tumors.

**Conclusions:**

These findings suggest that LAT1 mRNA is related to the invasive and progressive potential of clear cell RCC.

## Background

Renal cell carcinoma (RCC) is a common tumor that accounts for about 3% of all adult malignancies [1]. Localized RCC is generally considered to be suitable for surgical resection, but almost 30% of the patients with limited disease at the time of surgery develop metastasis within the next 3 years [2]. Furthermore, clear cell RCC is a highly vascular tumor, so many patients already have metastasis at the time of diagnosis [1]. Metastasis occurs when cancer cells spread from the primary tumor to distant sites [3], and is the major cause of cancer death. RCC patients with distant metastases have a poor prognosis and their 5-year survival rate is less than 10% [2].

Tumor cells require a steady and adequate supply of sugars and amino acids to maintain metabolism and protein synthesis at a high enough level for rapid growth and proliferation [4,5]. Aminoacid transporters are essential for the growth and proliferation of both normal cells and transformed cells [6,7]. The increased requirement of tumor cells for nutrients may be met by increasing the supply through vasculogenesis and by enhanced cellular uptake through upregulation of specific transporters [8]. The system large amino acid transporter (LAT) is a major nutrient transport system that is responsible for Na^+^-independent transport of large neutral amino acids [9,10]. It plays a critical role in the absorption of amino acids from the small intestine, as well as in movement of amino acids across the blood–brain barrier, the placenta, and the proximal tubules of the kidneys [6,7]. Interestingly, LAT1 is associated with cancerous or proliferative cells, and it has been reported that LAT1 is highly expressed in proliferating tissues, many tumor cell lines, and primary human tumors [10-16]. Thus, LAT1 may play a key role in the growth of tumor cells by promoting the uptake of essential amino acids. Indeed, the LAT1-specific inhibitor JPH203 (KYT0353) was reported to reduce the incorporation of essential amino acids by cancer cell lines and to attenuate the growth of human tumor cells implanted into nude mice [17], indicating that LAT1 might be an attractive target for cancer therapy.

After LAT1 was isolated by expression cloning, it was found to be co-expressed with the heavy chain of 4 F2 cell surface antigen (4F2hc) and to be involved in the transportation of neutral amino acids [9]. Three other LAT isoforms (LAT2, LAT3, and LAT4) have been identified in addition to LAT1 and together these four isoforms comprise the system L amino acid transporter [18-20]. The mRNAs of LAT2 and 4F2hc are ubiquitously expressed in normal tissues, including the glomerular parietal epithelial cells and podocytes in the kidney, and co-expression of LAT2 with 4F2hc promotes amino acid uptake as does the LAT1/4F2hc complex [18,21]. In addition, LAT3 has been localized to glomerular podocytes [22], while LAT4 is expressed in several organs such as the brain, intestine, placenta, and kidney [20]. In the kidney, LAT4 is found in the distal tubules and collecting ducts [20]. Thus, LATs 1–4 and 4F2hs seem to have an important influence on normal kidney function, but the expression and role of these proteins in human RCC remain unclear. Accordingly, this study was performed to investigate the expression of mRNAs for the four LATs (LAT1, LAT2, LAT3, and LAT4) and 4F2hc in RCC patients, and to compare the findings with clinicopathological data. It was hoped that the information thus obtained would shed light on the role of LATs in cancer progression.

## Methods

### Patients and samples

We studied 82 Japanese patients (62 men and 20 women) aged from 39 to 83 years (mean age: 63.1 years) who had newly diagnosed clear cell RCC (without sarcomatoid or rhabdoid components) from 1999 to 2012. All patients underwent CT and/or MRI for preoperative staging prior to radical nephrectomy. The postoperative follow-up period ranged from 3 to 112 months (median: 46 months). Surgery was performed before any other therapy. Patient and tumor characteristics are summarized in Table 1. In order to take into account possible inter-individual variation in the expression of LAT family (LAT1, LAT2, LAT3, LAT4, and 4F2hc) mRNAs and phosphorylated S6 ribosomal protein (Ser-235/236), tumor tissue samples and the corresponding non-tumor tissue samples obtained from the same patient were compared. The non-tumor control tissues were apparently free of RCC and were obtained from as distant a site as possible. If the tumor was located in the central part (middle portion) of the kidney, non-tumor tissues were extracted from the upper or lower pole. If the tumor was located in the upper or lower pole, non-tumor tissues were extracted from the opposite pole. The resected tissues were stored at -80°C, as described previously [23,24]. The tumor grade and clinical stage were determined according to the Fuhrman grading system and the TNM classification, respectively [25,26]. In the present study, all of the tumors were histological grades 1 to 3. Histopathological examination of the resected kidneys was performed independently by two pathologists. If abnormalities were later detected in the putatively normal tissue sample, the patient was excluded from the study. This study was conducted in accordance with the Helsinki Declaration and was approved by the Dokkyo Medical University Hospital institutional ethical review board. In addition, each patient signed a consent form that was approved by our institutional Committee on Human Rights in Research.

**Table 1:** Data collection of patient and tumor characteristics

**Patient**		
	No. of patients	82
	Age (yrs)	63.1 (39–83)
	Sex (male / female)	62 / 20
	follow-up times (months)	46 (3–112)
**Tumor**		
	Histological grading (G1 /G 2 / G3 / G4)	17 / 41 / 24 / 0
	pT stage (T1 / T2 / T3 / T4)	34 / 16 / 30 / 2
	Microscopic vascular invasion (v0 / v1)	49 / 33
	Metastasis (M0 / M1)	60 / 22

Postoperative adjuvant therapy with interferon (IFN)-alpha (3, 5, or 6 million units of natural human IFN-alpha two or three times a week), sorafenib (400 or 800 mg/day), or sunitinib (25 to 50 mg/day for 4 weeks, followed by two weeks of rest) was usually administered to patients with extra-renal involvement (metastatic disease) until progression occurred. The doses of these agents were decreased if grade 3/4 toxicity occurred.

### Real-time reverse transcription-polymerase chain reaction assay

Total RNA was purified from all 82 sets of tumor and non-tumor tissue samples with an RNA preparation kit (“High Pure RNA Kit”; Roche Diagnostic Ltd., Germany), and was used as a template for the synthesis of cDNA. The reaction mixture (100 μL) contained 1 μg of random hexamers and 100 units of MMLV-reverse transcriptase, with incubation being done at 25°C for 10 min, 42°C for 30 min, and then at 99°C for 5 min in a TP960 Thermal Cycler Dice (Takara Bio Ltd., Shiga, Japan) with SYBR Green. The following primers were used to amplify the indicated genes in tumor tissues after confirming their specificity (Takara Bio Ltd., Shiga, Japan)):

LAT1, sense; 5′- GCATCGGCTTCACCATCATC -3′,

anti-sense; 5′- ACCACCTGCATGAGCTTCTGAC -3′;

LAT2, sense; 5′- TTTGCCTATGGAGGCTGGAAC -3′,

anti-sense; 5′- GCGACATTGGCAAAGACATACAC -3′;

LAT3, sense; 5′- ATGGACTGGCGGATCAAGG -3′,

anti-sense; 5′- TCTTGCAGTAGCGTGGTCTGATG -3′;

LAT4, sense; 5′- TGCGTACGGAGCAAGTAAACCA -3′,

anti-sense; 5′- GAAGGTCATACACATCCCACCAAAG -3′;

4F2hc, sense; 5′- GGGTCCAATTCACAAGAACCAGA -3′,

anti-sense; 5′- TTGGGAGTAAGGTCCAGAATGACAC -3′; 7

β-actin, sense; 5′- CTGGCATCGTGATGGACTCCGG -3′,

anti-sense; 5′- GTGGATGCCACAGGACTCCATG-3′,.

Real-time RT-PCR was performed in a 25 μL reaction mixture containing 20 ng of sample cDNA, 100 nM sense primer, 100 nM anti-sense primer, and 12.5 μL of SYBR Green PCR Master Mix (Applied Biosystems). PCR was carried out with 45 cycles of 95°C for 15 sec and 60°C for 1 min. Then the products were normalized for β-actin as an internal control [14,15]. A standard curve was generated for each mRNA by five-fold dilution of a control RNA sample (25×, 5×, 1×, 0.2×, and 0.04×), and the expression of each target mRNA was calculated as a ratio to that of β-actin to determine the relative level of expression [23,24]. The mean value obtained by analyzing three samples of resected tissue was calculated as described previously [24].

### Western blotting

We could only perform Western blotting for 18 tumors. Samples of tumor tissue and normal tissue were carefully dissected free of stromal tissue. Western blotting for phosphorylated S6 ribosomal protein (Ser-235/236) was carried out as described previously [27,28]. In brief, 10 μg of cytosolic protein was separated by SDS-PAGE (4-12% gel), electrotransfer to a polyvinylidene difluoride membrane (iBlot Gel Transfer Stacks PVDF, Mini; Life Technologies, Carlsbad, CA) was performed. After the membrane was blocked, the bound proteins were probed with an anti-phosphorylated S6 ribosomal protein (Ser-235/236) antibody, 2 F9, which is an anti-human primary antibody and was raised in rabbits (Cell Signaling Technology, Inc; # 4856), and a primary antibody for β-actin (Millipore; # 1501R Bedford, MA). Hela cells were used as the positive control. Next, the membranes were washed and incubated with horseradish peroxidase-conjugated secondary antibodies. Bands of antibody-bound proteins were visualized by chemiluminescence, the blotted membrane was scanned for densitometry with a PDI imaging scanner (Agfa Japan, Tokyo), and the data were analyzed with NIH Image software (ImageJ for Mac OS, version 1.47). Expression of phosphorylated S6 ribosomal protein (Ser-235/236) was calculated relative to that of β-actin in the tumor tissue specimens and corresponding normal tissue specimens. For quantification of these proteins, the relative amount of phosphorylated S6 ribosomal protein (Ser-235/236) in tumor tissue was expressed as a ratio of the optical density of the band for the tumor tissue specimen to that for the corresponding normal tissue specimen (set at 1.0) by densitometric analysis, as described previously [27,28]. The mean values for specimens of tumor and non-tumor tissue were calculated from three experiments [27,28].

### Statistical analysis

Comparison between groups was performed by the Mann–Whitney *U*-test for two groups (pT stage, microscopic vascular invasion, and metastasis) or the Kruskal-Wallis test for three groups (tumor histological grade), as described previously [13-15]. Spearman’s rank correlation coefficient analysis was employed to determine the relation between LAT1 mRNA and phosphorylated S6 ribosomal protein (Ser-235/236) expression. LAT mRNA expression, tumor grade, pT stage, microscopic vascular invasion, and metastasis were assessed for their impact on survival by Cox proportional hazards analysis using univariate and multivariate models. The Kaplan-Meier method was employed to estimate survival, for various groups, and differences between the groups were assessed by the log-rank test. In all analyses, a probability (*P*) value of less than 0.05 was considered to indicate significance. Data were analyzed with commercially available software.

## Results

### LATs mRNAs expression and tumor characteristics

**Figure 1:**
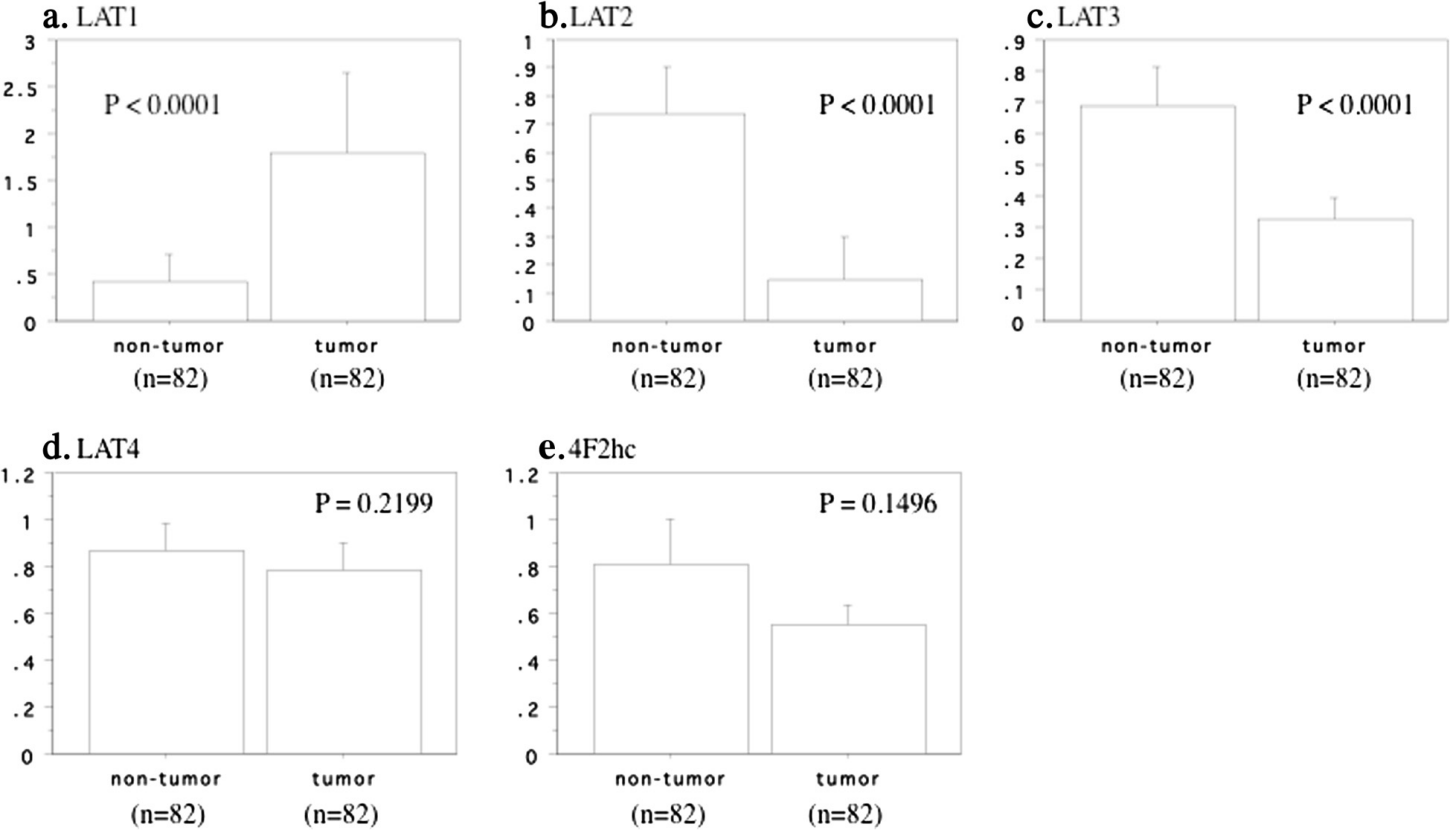
**LATs mRNA expressions between tumor and non-tumor tissues in human clear cell renal cell carcinoma. a**; LAT1, **b**; LAT2, **c**; LAT3, **d**; LAT4, **e**; 4F2hc. The data show the 95% confidential interval.

Although the expression of LAT1 mRNA was increased in tumor tissue (mean ± S.D. = 1.78 ± 3.95) compared with non-tumor tissue (0.42 ± 1.36, *P* < 0.0001, Figure 1A), expression of LAT2 and LAT3 mRNAs was decreased in the tumors (0.14 ± 0.72 versus 0.74 ± 0.76, *P* < 0.0001, Figure 1B, and 0.32 ± 0.31 versus 0.69 ± 0.55, *P* < 0.0001, Figure 1C, respectively). In contrast, there were no differences of LAT4 and 4F2hc mRNA expression between tumor and non-tumor tissues (LAT4: 0.79 ± 0.53 versus 0.87 ± 0.53, *P* = 0.2199, Figure 1D; 4F2hc: 0.55 ± 0.39 versus 0.81 ± 0.85, *P* = 0.1496, Figure 1E).

**Figure 2:**
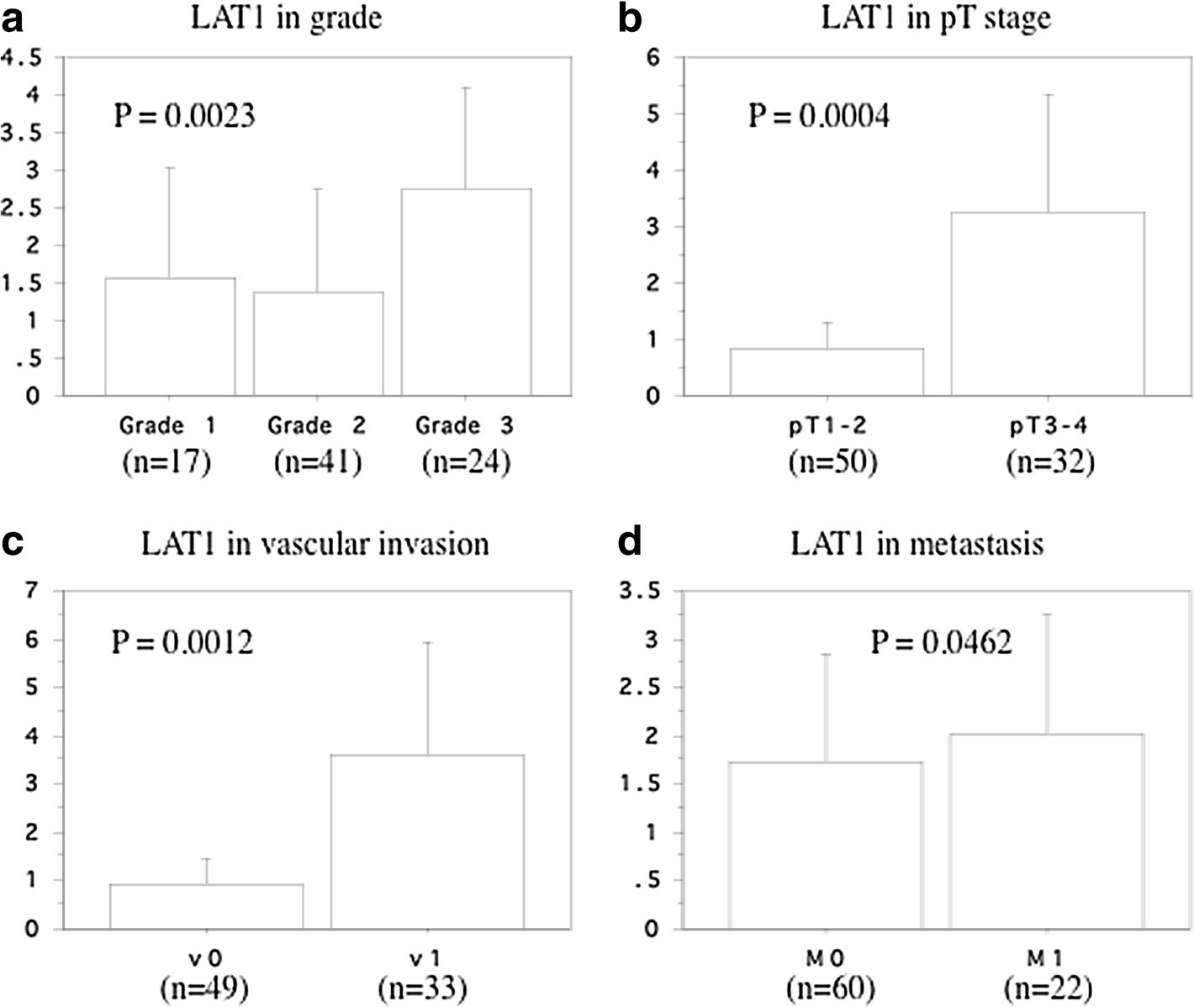
**LAT1 mRNAs in tumors. a**; grade. **b**; pT stage. **c**; microscopic vascular invasion. **d**; metastasis. The data show the 95% confidential interval.

Increased expression of LAT1 mRNA in primary renal tumors was related to the poorer differentiation (Figure 2A, Table 2). Expression in primary renal tumors was not related to the histological grade in the case of LAT2 mRNA, LAT3 mRNA, and LAT4 mRNA as well as 4F2hc mRNA (Table 2).

**Table 2:** Quantitative PCR measurements of LATs mRNAs and pS6K protein densitometry results

		**Real time RT-PCR for mRNAs**											**Western blotting for pS6K protein**		
			**LAT1**		**LAT2**		**LAT3**		**LAT4**		**4h2Fc**		**Number = 18**		
		**Number = 82**		***P*** **value**		***P*** **value**		***P*** **value**		***P value***		***P value***			**Number = 18**
Tissue	tumor	n = 82	1.78 ± 3.95	< 0.0001	0.14 ± 0.72	< 0.0001	0.32 ± 0.31	< 0.0001	0.79 ± 0.53	0.2199	0.55 ± 0.39	0.1496	n = 18	3.03 ± 1.91	3.03 ± 1.91
	non-tumor	n = 82	0.42 ± 1.36		0.74 ± 0.76		0.69 ± 0.55		0.87 ± 0.53		0.81 ± 0.85		n = 18	1.27 ± 0.37	
Grade	G1	n = 17	1.56 ± 2.33	0.0023	0.04 ± 0.03		0.29 ± 0.29	0.6275	0.87 ± 0.45	0.1214	0.62 ± 0.38	0.6340	n = 3	3.84 ± 2.25	0.3596
	G2	n = 41	1.36 ± 4.61		0.07 ± 0.07	0.9112	0.35 ± 0.36		0.90 ± 0.59		0.55 ± 0.38		n = 9	2.63 ± 1.66	
	G3	n = 24	2.75 ± 3.18		0.34 ± 1.32		0.30 ± 0.20		0.55 ± 0.36		0.54 ± 0.43		n = 6	4.27 ± 1.99	
pT stage	pT1-2	n = 50	0.85 ± 1.58	0.0004	0.06 ± 0.06	0.556	0.34 ± 0.36	0.9583	0.85 ± 0.55	0.1103	0.54 ± 0.37	09280	n = 10	2.48 ± 1.12	0.0849
	pT3-4	n = 32	3.24 ± 5.76		0.28 ± 1.15		0.30 ± 0.20		0.68 ± 0.47		0.57 ± 0.43		n = 8	3.64 ± 2.21	
Microscopic vascular invasion	v0	n = 49	0.88 ± 1.60	0.0012	0.06 ± 0.06	0.7122	0.31 ± 0.34	0.3869	0.79 ± 0.50	0.6330	0.55 ± 0.40	0.9582	n = 10	2.77 ± 1.12	0.2087
	v1	n = 33	3.11 ± 5.70		0.27 ± 1.13		0.35 ± 0.26		0.78 ± 0.57		0.55 ± 0.39		n = 8	3.66 ± 2.33	
Metastasis	M0	n = 60	1.73 ± 4.34	0.0462	0.08 ± 0.13	0.5461	0.34 ± 0.22	0.4314	0.83 ± 0.53	0.6125	0.58 ± 0.29	0.3429	n = 9	2.11 ± 1.24	0.0083
	M1	n = 22	2.02 ± 2.69		0.17 ± 0.84		0.32 ± 0.33		0.78 ± 0.53		0.55 ± 0.42		n = 9	3.84 ± 2.05	

Data show mean ± S.D.

A higher level of LAT1 mRNA expression in the primary tumor was associated with local invasion (Figure 2B, Table 2). Expression of LAT2 and LAT3 mRNAs was lower in tumor tissue than in non-tumor tissue, and neither LAT2 nor LAT3 was associated with local invasion (Table 2). Expression of LAT4 and 4F2hc mRNAs in the primary tumor was also unrelated to the pT stage (Table 2).

Higher expression of LAT1 mRNA in the primary tumor was associated with microscopic vascular invasion (Figure 2C, Table 2). In contrast, expression of the other LAT mRNAs showed no difference between v1 and v0 tumors (Table 2).

Investigation of the association with metastasis showed that the level of LAT1 mRNA expression in primary tumor tissues differed significantly between RCC with metastasis (M1) or without metastasis (M0) (Figure 2D, Table 2). In contrast, there was no difference in the expression of LAT2, LAT3, or LAT4 mRNAs, as well as 4F2hc mRNA (Table 2).

### Relationship between LAT mRNA and phosphorylated S6 ribosomal protein (Ser-235/236)

**Figure 3:**
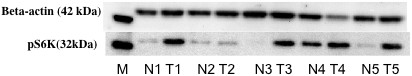
**Expression of phosphorylated S6 ribosomal protein (Ser-235/236) (32 kDa) and beta actin (42 kDa) proteins in the primary tumor tissues using Western blotting.** M; marker. N; non-tumor tissue. T; primary tumor tissue with metastatic lesions. Each number corresponds to a case number.

**Figure 4:**
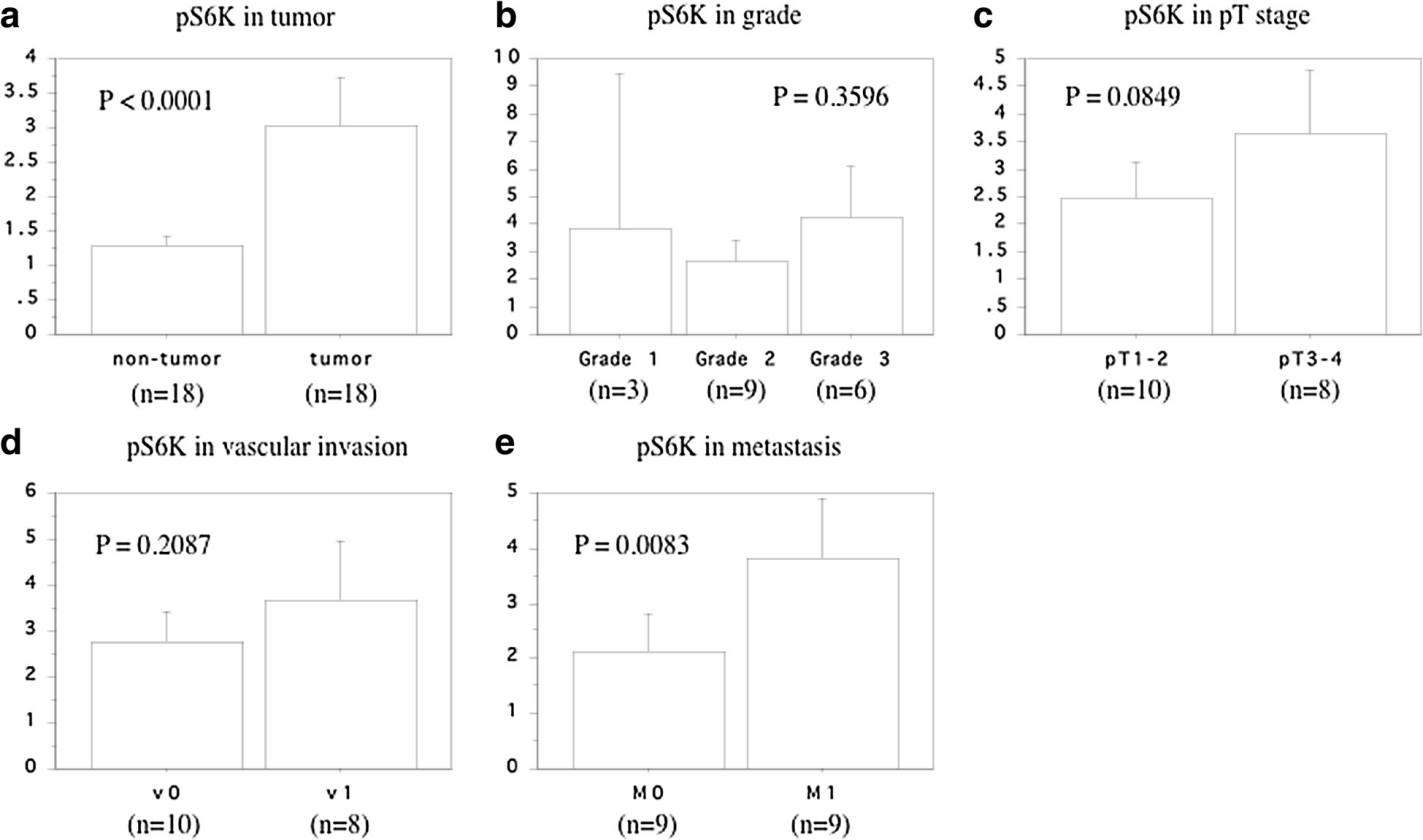
**Expression of phosphorylated S6 ribosomal protein (Ser-235/236). a**; tumor. **b**; grade. **c**; pT stage. **d**; microscopic vascular invasion. **e**; metastasis. The data show the 95% confidential interval.

**Figure 5:**
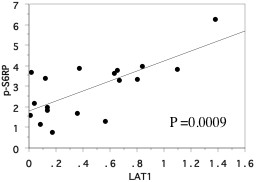
**Spearman rank correlation coefficient relationship.** X axis is an independent variable. Y axis is a dependent variable. LAT1 mRNA levels positively correlated with phosphorylated S6 ribosomal protein (Ser-235/236) levels in primary tumor tissues.

Western blotting of 18 resected kidney specimen showed that the expression of phosphorylated S6 ribosomal protein (Ser-235/236) was higher in primary tumors than in normal tissues (Figure 3, Table 2), and its increased expression was associated with metastasis, but not grade, pT stage, and vascular invasion (Figure 4, Table 2). We investigated the correlation between LAT1 mRNA and phosphorylated S6 ribosomal protein (Ser-235/236) expression in 18 tumor tissues. When LAT1 was used as an independent variable and phosphorylated S6 ribosomal protein (Ser-235/236) as a dependent variable, a positive correlation between them was observed (r2 = 0.508, *P* = 0.0009, Figure 5).

### LATs mRNAs expression and survival

**Figure 6:**
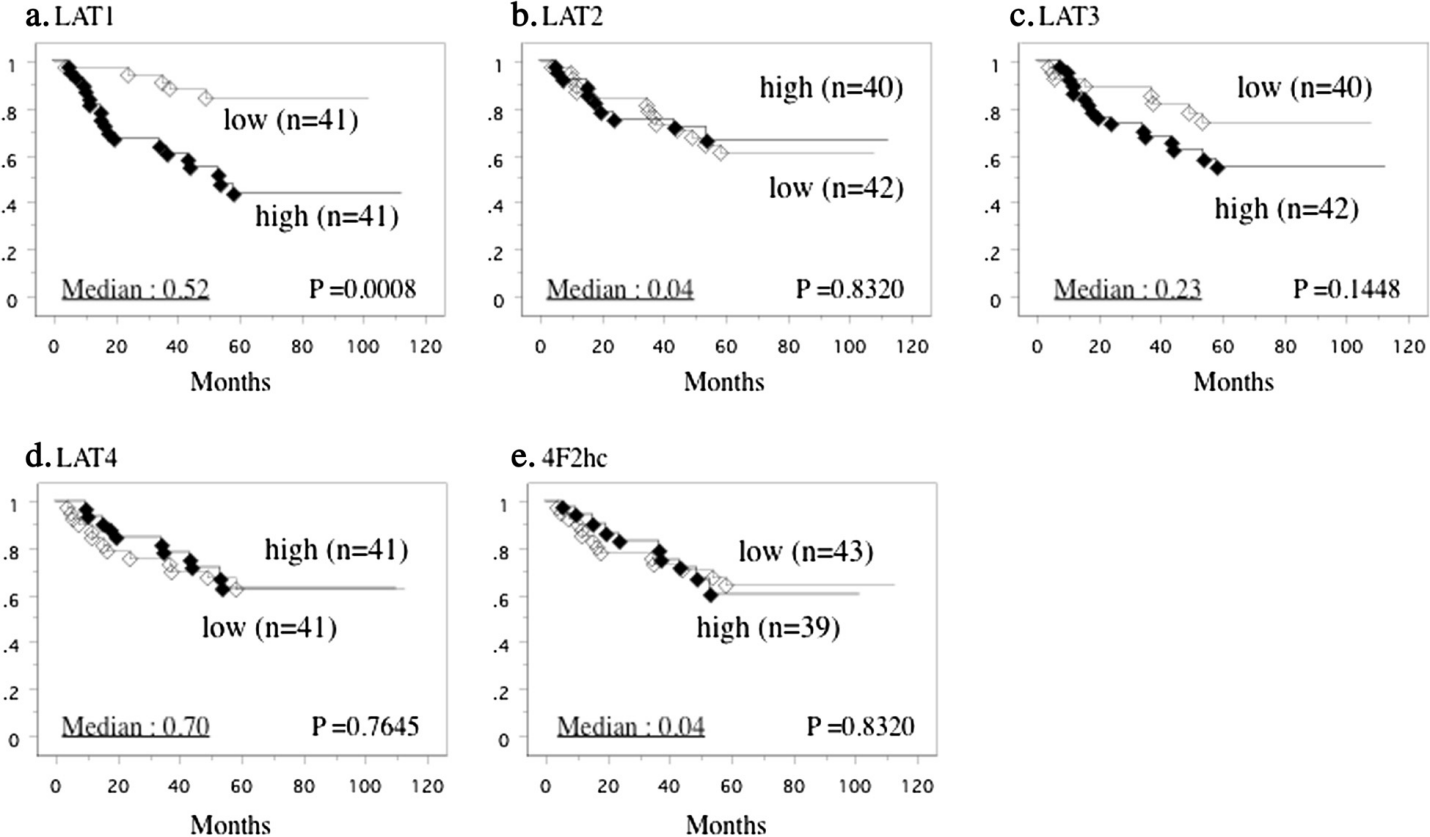
**Survival curve based on the median values of mRNA expression of LATs in tumors, the cases were divided into two groups at this levels high and low expression. a**; LAT1, **b**; LAT2, **c**; LAT3, **d**; LAT4, **e**; 4F2hc.

The median level of L expression in tumor tissues was 0.52, so the patients were divided into two groups at this cut-off value to give a high-expression group (n = 41) and a low-expression group (n = 41). Kaplan-Meier plots of survival for the high-expression and low-expression groups showed that increased expression of LAT1 mRNA was associated with shorter overall survival (*P* = 0.0008, Figure 6A). In contrast, on the similar criteria as well as LAT1, the levels of the other LAT mRNAs were not related to overall survival (Figures 6B-E).

Univariate analysis of overall survival was performed with the Cox proportional hazards model and it revealed that histological grade, pT stage, microscopic vascular invasion, metastasis, and LAT1 mRNA expression were all significant determinants of survival. On multivariate analysis, metastasis was identified as an independent factor (*P* = 0.0172) for survival and pT stage showed a weak association (*P* = 0.0616) (Table 3).

**Table 3:** Cox regression analysis for various potential prognostic factors in survival

**Overall survival in all patients**						
**Variable**	**Unfavorable/favorable characteristics**	**No. of patients**	**Analysis**	**Relative risk**	**95% confidential interval**	**P value**
			Univariate (U)	6.080	2.799 - 13.209	< 0.0001
**Grade**	3 / 2 / 1	21 / 33 / 17				
			Multivariate (M)	1.693	0.838 - 3.424	0.1424
			U	41.532	5.598 - 308.114	< 0.0001
**pT**	4,3 / 2,1	43 / 28				
			M	9.21	0.897 - 94.539	0.0616
			U	9.981	3.407 - 29.245	< 0.0001
**v**	(+) / (−)	43 / 28				
			M	1.548	0.423 - 5.674	0.5093
			U	12.098	4.927 - 29.709	< 0.0001
**M**	(+) / (−)	18 / 53				
			M	3.222	1.231 - 8.434	0.0172
			U	4.625	1.724 - 12.407	0.0023
**LAT1**	high / low	41 / 41				
			M	1.977	0.635 - 6.150	0.2393

## Discussion and conclusions

To the best of our knowledge, this is the first investigation of the relation between the expression of LAT mRNAs (LAT1, LAT2, LAT3, and LAT4) or 4h2hc mRNA and the clinicopathologic features of clear cell RCC. To allow for possible inter-individual variation in the expression of LAT mRNAs, we performed comparison of mRNA expression between paired samples of tumor and non-tumor tissues from the same kidney. This revealed that LAT1 mRNA expression was higher in tumor tissue than in non-tumor tissue. In addition, the LAT1 mRNA level was significantly higher in less differentiated primary tumors (grade 3), as well as tumors with local invasion (pT3-4), microscopic vascular invasion (v1), and metastasis (M1), than in tumors without these features. Furthermore, increased expression of LAT1 mRNA in the primary tumor was correlated with an unfavorable prognosis. These findings suggest that LAT1 may have an influence on the invasive potential and progression of clear cell RCC.

The primary features of the malignant phenotype are maintained via intrinsic modification of metabolic activity, which is characterized by enhancement of the nutrient supply, energy production, and synthesis of a variety of macromolecular components. This metabolic shift in transformed cells, as compared with non-proliferating cells, involves aberrant activation of aerobic glycolysis, de novo lipid biosynthesis, and glutamine-dependent anaplerosis to fuel rapid cell growth and proliferation. [4,5]. Conversion of glucose metabolism from oxidation to glycolysis (the Warburg effect) is one of the typical strategies employed for the generation of ATP by cancer cells [29]. Because tumor cells have an increased requirement for nutrients, this is met by increasing nutrient availability through vasculogenesis and by enhanced cellular uptake of nutrients through upregulation of specific transporters [8]. Given this well-established influence of energy metabolism on tumor development and growth, reprogramming of energy metabolism can be viewed as one of the “Hallmarks of Cancer” [30].

Amino acids are essential for protein synthesis, and thus are required for the growth and proliferation of both normal and transformed cells. Amino acid transport across the plasma membrane is mediated by various amino acid transporters that are localized to the membrane [6,7]. Among them, LAT is a major nutrient transport system that contributes to the growth and proliferation of both normal and transformed cells [6,31]. LAT is also essential for amino acid transport in the proximal tubules of the kidneys [6,7], and clear cell RCC has been suggested to arise from the proximal tubules [32].

LAT1 was the first LAT isoform to be isolated, and it has been reported that LAT1 is overexpressed in primary human neoplasms and involved in tumor cell proliferation due to its role in the transport of essential amino acids [10,33]. There is evidence that increased LAT1 expression is associated with a poor prognosis of various cancers, including brain tumors [11], lung cancer [12], gastric cancer [13], urothelial cancer [14], and prostatic cancer [15]. Furthermore, it has been reported that LAT1 not only provides cancer cells with amino acids required for protein synthesis but also with amino acids that stimulate cell growth via mammalian targeting of rapamycin (mTOR) [31], and that the amino acid supply is coupled to cell signaling via mTOR in mammalian cells and influences both cell growth and cell cycle progression [34,35]. Wang et al. recently reported that prostate cancer cells regulate LAT1 expression to maintain sufficient levels of leucine for mTOR complex 1 (mTORC1) signaling and cell growth, while inhibiting LAT function led to decreased growth and mTORC1 signaling in these cells [36]. Thus, mTORC1 controls cell growth by regulating protein synthesis, and is a potential antitumor target and mTOR inhibitors are currently under investigation for the treatment of various human cancers. mTORC1 lies downstream of PI3K/Akt pathway and this pathway is frequently activated in human clear cell RCCs [28], so mTORC1 represents a pivotal target for anticancer therapy in RCCs [37-39]. In our previous report, phosphorylated S6 ribosomal protein (Ser-235/236), the best-characterized downstream effector of mTORC1, was upregulated in the primary tumors with metastatic phenotype [28]. In the present study, the tumor tissue levels of LAT1 mRNA and phosphorylated S6 ribosomal protein (Ser-235/236) were positively correlated, and higher expression level of LAT1 mRNA and phosphorylated S6 ribosomal protein (Ser-235/236) was associated with metastatic potential. Taken together with these reports, our findings suggest that LAT1 and phosphorylated S6 ribosomal protein (Ser-235/236) may cooperatively influence the invasive potential and progression of RCC.

On the other hand, how the LATs are associated with cancer has not been fully elucidated from the molecular biological perspective. Hayashi et al. recently reported that c-Myc is crucial for the expression of LAT1, and LAT1 is a central transporter of essential neutral amino acids in human pancreatic cancer cells [40]. c-Myc is a proto-oncogene that encodes a transcription factor, and it is known to enhance biosynthesis as well as energy generation, with genes involved in glucose transport and the glycotic pathway being upregulated by c-Myc [41,42]. Recently, closer attention has been paid to the role of Myc in cancer cell metabolism for cancer treatment [43,44]. On the other hand, several studies have shown that the c-Myc pathway is activated in RCC due to overexpression and amplification of the c-Myc gene. [45,46]. Thus, c-Myc might play a role in tumorigenesis by regulating the expression of genes involved in metabolism that are required for cell proliferation and development of the malignant phenotype.

In the present study, RCC showed lower expression of LAT2 and LAT3 mRNAs in comparison with non-tumor renal tissue. In contrast, there were no differences in the expression of LAT4 and 4F2hc mRNAs. Luo et al. reported that the level of LAT2 mRNA, but not 4F2hc mRNA, was significantly higher in leiomyoma tissue compared with matched myometrial tissue, and that small interfering RNA knockdown of LAT2 or 4F2hc markedly increased the growth of primary human uterine leiomyoma smooth muscle cells, indicating that LAT2/4F2hc may play an important role in leiomyoma cell proliferation [47]. Kaira et al. recently reported that 4F2hc expression increased from a low to high histological grade and was significantly associated with worse overall survival in patients with pulmonary neuroendocrine tumors [48]. 4F2hc has been reported to be involved in cellular proliferation, transformation, fusion, and adhesion, and it also contributes to the LAT system. In addition, 4F2hc is involved in regulating integrin activation, and therefore has a role in integrin signaling and anchorage-independent growth. 4F2hc is reconstituted and expressed at high levels on the surface of many types of tumor cells. Recent studies have demonstrated that 4F2hc expression is increased in a variety of cancers and has a crucial role in the progression and metastasis of human neoplasms [49-51]. In contrast to the above, there have been few reports about the expression of LAT3 and LAT4 mRNAs in human cancer.

The present study revealed that increased LAT1 mRNA expression is associated with invasion of RCC and an unfavorable prognosis, suggesting a potential role of LAT1 upregulation in the progression of human cancer and the possibility of using LAT1 mRNA as a target for anticancer treatment. However, our study included a relatively small number of patients and the follow-up period was too short to draw definite conclusions regarding the possible relations between LAT mRNAs and the prognosis of RCC. Moreover, it is important to study the relationship between expression of LAT mRNAs and the efficacy of IFN-alpha, sorafenib, and sunitinib. Furthermore, we should investigate the molecules transported by LATs that are key players in carcinogenesis and cancer progression in order to fully elucidate the molecular mechanisms by which LATs participate in human diseases including cancer. Such information may shed light on the LAT mRNAs that are useful biomarkers.

## Competing interests

The authors declare that they have no competing interests.

## Authors’ contributions

HB, TF and TK* initiated the study, participated in its design and coordination, carried out the study, performed the statistical analysis. HB, TF and TK* drafted the manuscript. DN, TM, HY, AM, YY, HA, MY and YF carried out the study. NA and K-IY participated in the design of the study and helped to draft the manuscript. All authors read and approved the final manuscript.

## Pre-publication history

The pre-publication history for this paper can be accessed here:

http://www.biomedcentral.com/1471-2407/13/509/prepub

## References

[B1] Motzer RJ Bander NH Nanus DM Renal-cell carcinoma N Engl J Med 1996 335 865 875 10.1056/NEJM199609193351207 8778606

[B2] Athar U Gentile TC Treatment options for metastatic renal cell carcinoma: a review Can J Urol 2008 15 523 532 18405442

[B3] Chaffer CL Weinberg RA A perspective on cancer cell metastasis Science 2011 331 1559 1564 10.1126/science.1203543 21436443

[B4] De Berardinis RJ Julian JL Hatzivassiliou G Thompson CB The biology of cancer: metabolic reprogramming fuelscell growth and proliferation Cell Metab 2008 7 11 20 10.1016/j.cmet.2007.10.002 18177721

[B5] Barger JF Plas DR Balancing biosynthesis and bioenergetics: metabolic programs in oncogenesis Endocr Relat Cancer 2010 17 R287 R304 10.1677/ERC-10-0106 20699334

[B6] Christensen HN Role of amino acid transport and countertransport in nurtrition and metabolism Physiol Rev 1990 70 43 77 2404290 10.1152/physrev.1990.70.1.43

[B7] McGivan JD Pastor-Anglada M Regulatory and molecular aspects of mammalian amino acid transport Biochem J 1994 299 321 334 8172590 10.1042/bj2990321 PMC1138275

[B8] Ganapathy V Thangaraju M Prasad PD Nutrient transporters in caner: relevance to Warburg hypothesis and beyond Pharmacol THer 2009 121 29 40 10.1016/j.pharmthera.2008.09.005 18992769

[B9] Kanai Y Segawa H Miyamoto K Uchino H Takeda E Endou H Expression cloning and characterization of a transporter for large neutral amino acids activated by the heavy chain of 4 F2 antigen (CD98) J Biol Chem 1998 273 23629 23632 10.1074/jbc.273.37.23629 9726963

[B10] Yanagida O Kanai Y Chairoungdua A Kim DK Segawa H Nii T Cha SH Matsuo H Fukushima J Fukasawa Y Tani Y Taketani Y Uchino H Kim JY Inatomi J Okayasu I Miyamoto K Takeda E Goya T Endou H Human L-type amino acid transporter 1 (LAT1): characterization of function and expression in tumor cell lines Biochim Biophys Acta 2001 1514 291 302 10.1016/S0005-2736(01)00384-4 11557028

[B11] Kobayashi H Ishii Y Takayama T Expression of L-type amino acid transporter 1 (LAT1) in esophageal carcinoma J Surg Oncol 2005 90 233 238 10.1002/jso.20257 15906366

[B12] Nawashiro H Otani N Shinomiya N Fukui S Ooigawa H Shima K Matsuo H Kanai Y Endou H L-type amino acid transporter 1 as a potential molecular target in human astrocytic tumors Int J Cancer 2006 119 484 492 10.1002/ijc.21866 16496379

[B13] Kaira K Oriuchi N Imai H Shimizu K Yanagitani N Sunaga N Hisada T Tanaka S Ishizuka T Kanai Y Endou H Nakajima T Mori M Prognostic significance of L-type amino acid transporter 1 expression in resectable stage I-III nonsmall cell lung cancer Br J Cancer 2008 98 742 748 10.1038/sj.bjc.6604235 18253116 PMC2259171

[B14] Ichinoe M Mikami T Yoshida T Igawa I Tsuruta T Nakada N Anzai N Suzuki Y Endou H Okayasu I High expression of L-type amino acid transporter 1 (LAT1) in gastric carcinomas: comparison with non-cancerous lesions Pathol Int 2011 61 281 289 10.1111/j.1440-1827.2011.02650.x 21501294

[B15] Nakanishi K Ogata S Matsui H Kanai Y Endou H Hiroi S Tominaga S Aida S Kasamatsu H Kawai T Expression of LAT1 predicts risk of progression of transitional cell carcinoma of the upper urinary tract Virchows Arch 2007 451 681 690 10.1007/s00428-007-0457-9 17622555

[B16] Sakata T Ferdous G Tsuruta T Satoh T Baba S Muto T Ueno A Kanai Y Endou H Okayasu I L-type amino acid transporter 1 as a novel biomarker for high-grade malignancy in prostate cancer Pathol Int 2009 59 7 18 10.1111/j.1440-1827.2008.02319.x 19121087

[B17] Oda K Hosoda N Endo H Saito K Tsujihara K Yamamura M Sakata T Anzai N Wempe MF Kanai Y Endou H L-type amino acid transporter 1 inhibitors inhibit tumor cell growth Cancer Sci 2010 101 173 179 10.1111/j.1349-7006.2009.01386.x 19900191 PMC11158286

[B18] Wagner CA Lang F Broer S Function and structure of heterodimeric acid transporters Am J Physio Cell Physiol 2001 281 c1077 c1093 10.1152/ajpcell.2001.281.4.C1077 11546643

[B19] Babu E Kanai Y Chairoungdua A Kim DK Iribe Y Tangtrongsup S Jutabha P Li Y Ahmed N Sakamoto S Anzai N Nagamori S Endou H Identification of a novel system L amino acid transporter structurally distinct from heterodimeric amino acid transporters J Biol Chem 2003 278 43838 43845 10.1074/jbc.M305221200 12930836

[B20] Bodoy S Martín L Zorzano A Palacín M Estévez R Bertran J Identification of LAT4, a novel amino acid transporter with system L activity J Biol Chem 2005 280 12002 12022 10.1074/jbc.M408638200 15659399

[B21] Kurayama R Ito N Nishibori Y Fukuhara D Akimoto Y Higashihara E Ishigaki Y Sai Y Miyamoto K Endou H Kanai Y Yan K Role of amino acid transporter LAT2 in the activation of mTORC1 pathway and the pathogenesis of crescentic glomerulonephritis Lab Invest 2011 91 992 1006 10.1038/labinvest.2011.43 21403644

[B22] Sekine Y Nishibori Y Akimoto Y Kudo A Ito N Fukuhara D Kurayama R Higashihara E Babu E Kanai Y Asanuma K Nagata M Majumdar A Tryggvason K Yan K Amino acid transporter LAT3 is required for podocyte development and function J Am Soc Nephrol 2009 20 1586 1596 10.1681/ASN.2008070809 19443642 PMC2709689

[B23] Suwa H Ohshio G Imamura T Watanabe G Arii S Imamura M Narumiya S Hiai H Fukumoto M Overexpression of the *rho*C gene correlates with progression of ductal adenocarcinoma of the pancreas Br J Cancer 1998 77 147 152 10.1038/bjc.1998.23 9459160 PMC2151257

[B24] Kamai T Yanai Y Arai K Abe H Yamanishi T Kurimoto M Yoshida K-I Increased interferon alpha receptor 2 mRNA levels is associated with renal cell carcinoma metastasis BMC Cancer 2007 7 1471 2407 7-159 10.1186/1471-2407-7-159 PMC1988828 17697365

[B25] Fuhrman SA Lasky LC Lmas C Prognostic significance of morphologic parameters in renal cell carcinoma Am J Surg Pathol 1982 6 655 663 10.1097/00000478-198210000-00007 7180965

[B26] Sobin LH Gospodarowicz MK Wittekind CH International union against cancer. UICC TNM classification of malignant tumors 2009 7 New York: Wiley-Liss 255 257

[B27] Kamai T Tsujii T Arai K Takagi K Asami H Ito Y Oshima H Yoshida K-I Significant association of Rho/ROCK pathway with invasion and metastasis of bladder cancer Clin Cancer Res 2003 9 2632 2641 12855641

[B28] Furuya N Kamai T Shirataki H Yanai Y Fukuda T Mizuno T Kambara T Nakanishi K Abe H Yoshida K-I Serum interferon alpha receptor 2 mRNA may predict efficacy of interferon alpha with/without low-dose sorafenib for metastatic clear cell renal cell carcinoma Cancer Immunol Immunother 2011 60 793 808 10.1007/s00262-011-0989-3 21350947 PMC3098978

[B29] Vander Heiden MG Cantley LC Thompson CB Understanding the Warburg effect: the metabolic requirements of cell proliferation Science 2009 324 1029 1033 10.1126/science.1160809 19460998 PMC2849637

[B30] Hanahan D Weinberg RA Hallmarks of cancer: the next generation Cell 2011 144 646 674 10.1016/j.cell.2011.02.013 21376230

[B31] Oxender DL Christensen HN Evidence for two types of mediation of neutral amino acid transport in Ehrlich cells Nature 1963 197 765 767 10.1038/197765a0 13940861

[B32] Novick AC Wein AJ, Kavoussi LR, Novick AC, Partin AW, Peters CA Open surgery of the kidney Campbell-Walsh Urology 2007 9 Philadelphia: Saunders Elsevier 1686 1758

[B33] Fuchs BC Bode BP Amino acid transporters ASCT2 and LAT1 in cancer: partners in crime? Semin Cancer Biol 2005 15 254 266 10.1016/j.semcancer.2005.04.005 15916903

[B34] Yamauchi K Sakurai H Kimura T Wiriyasermkul P Nagamori S Kanai Y Kohno N System L amino acid transporter inhibitor enhances anti-tumor activity of cisplatin in a head and neck squamous cell carcinoma cell line Cancer Lett 2009 276 95 101 10.1016/j.canlet.2008.10.035 19058911

[B35] Imai H Kaira K Oriuchi N Shimizu K Tominaga H Yanagitani N Sunaga N Ishizuka T Nagamori S Promchan K Nakajima T Yamamoto N Mori M Kanai Y Inhibition of L-type amino acid transporter 1 has antitumor activity in non-small cell lung cancer Anticancer Res 2010 30 4819 4828 21187458

[B36] Wang Q Bailey CG Ng C Tiffen J Thoeng A Minhas V Lehman ML Hendy SC Buchanan G Nelson CC Rasko JE Holst J Androgen receptor and nutrient signaling pathways coordinate the demand for increased amino acid transport during prostate cancer progression Cancer Res 2011 71 7525 7536 10.1158/0008-5472.CAN-11-1821 22007000

[B37] Abe H Kamai T Recent advances in the treatment of metastatic renal cell carcinoma Int J Urol 2013 10.1111 In publication 10.1111/iju.12187 23692504

[B38] Hudes G Carducci M Tomczak P Dutcher J Figlin R Kapoor A Staroslawska E Sosman J McDermott D Bodrogi I Kovacevic Z Lesovoy V Schmidt-Wolf IG Barbarash O Gokmen E O’Toole T Lustgarten S Moore L Motzer RJ Global ARCC trial : temsirolimus, interferon Alfa, or both for advanced renal-cell carcinoma N Engl J Med 2007 356 2271 2281 10.1056/NEJMoa066838 17538086

[B39] Motzer RJ Escudier B Oudard S Hutson TE Porta C Bracarda S Grünwald V Thompson JA Figlin RA Hollaender N Urbanowitz G Berg WJ Kay A Lebwohl D Ravaud A RECORD-1 study group: efficacy of everolimus in advanced renal cell carcinoma: a double-blind, randomised, placebo-controlled phase III trial Lancet 2008 372 449 456 10.1016/S0140-6736(08)61039-9 18653228

[B40] Hayashi K Jutabha P Endou H Anzai N c-Myc is crucial for the expression of LAT1 in MIA paca-2 human pancreatic cancer cells Oncol Rep 2012 28 862 866 22736142 10.3892/or.2012.1878

[B41] Kim JW Zeller KI Wang Y Jegga AG Aronow BJ O’Donnell KA Dang CV Evaluation of myc E-box phylogenetic footprints in glycolytic genes by chromatin immunoprecipitation assays Mol Cell Biol 2004 24 5923 5936 10.1128/MCB.24.13.5923-5936.2004 15199147 PMC480875

[B42] Osthus RC Shim H Kim S Li Q Reddy R Mukherjee M Xu Y Wonsey D LA LA Dang CV Deregulation of glucose transporter 1 and glycolytic gene expression by c-Myc J Biol Chem 2000 275 21797 21800 10.1074/jbc.C000023200 10823814

[B43] Dang CV Le A Gao P MYC-induced cancer cell energy metabolism and therapeutic opportunities Clin Cancer Res 2009 15 6479 6483 10.1158/1078-0432.CCR-09-0889 19861459 PMC2783410

[B44] Dang CV Rethinking the Warburg effect with Myc micromanaging glutamine metabolism Cancer Res 2010 70 859 862 10.1158/0008-5472.CAN-09-3556 20086171 PMC2818441

[B45] Tang SW Chang WH Su YC Chen YC Lai YH Wu PT Hsu CI Lin WC Lai MK Lin JY MYC pathway is activated in clear cell renal cell carcinoma and essential for proliferation of clear cell renal cell carcinoma Cancer Lett 2009 273 35 43 10.1016/j.canlet.2008.07.038 18809243

[B46] Liu Y Yin B Zhang C Zhou L Fan J Has-let-7a functions as a tumor suppressor in renal cell carcinoma cell lines by targeting c-myc Biochem Biophys Res Commun 2012 417 371 375 10.1016/j.bbrc.2011.11.119 22155254

[B47] Luo X Yin P Reierstad S Ishikawa H Lin Z Pavone ME Zhao H Marsh EE Bulun SE Progesterone and Mifepristone regulate L-type amino acid transporter 2 and 4 F2 heavy chain expression in uterine leiomyoma cells J Clin Endocrinol Metab 2009 94 4533 4539 10.1210/jc.2009-1286 19808856 PMC2775649

[B48] Kaira K Ohde Y Endo M Nakagawa K Okumura T Takahashi T Murakami H Tsuya A Nakamura Y Naito T Kondo H Nakajima T Yamamoto N Expression of 4F2hc (CD98) in pulmonary neuroendocrine tumors Oncol Rep 2011 26 931 937 21750865 10.3892/or.2011.1384

[B49] Takeuchi H Kubota T Kitai R Nakagawa T Hashimoto N CD98 immunoreactivity in multinucleated giant cells of glioblastomas: an immunohistochemical double labeling study Neuropathology 2008 28 127 131 10.1111/j.1440-1789.2007.00859.x 18021193

[B50] Kaira K Oriuchi N Shimizu K Ishikita T Higuchi T Imai H Yanagitani N Sunaga N Hisada T Ishizuka T Kanai Y Endou H Nakajima T Endo K Mori M Correlation of angiogenesis with 18 F-FMT and 18 F-FDG uptake in non-small cell lung cancer Cancer Sci 2009 100 753 758 10.1111/j.1349-7006.2008.01077.x 19141127 PMC11158756

[B51] Kaira K Oriuchi N Imai H Shimizu K Yanagitani N Sunaga N Hisada T Kawashima O Kamide Y Ishizuka T Kanai Y Nakajima T Mori M CD98 expression is associated with poor prognosis in resected non-small cell lung cancer with lymph node metastasis Ann Surg Oncol 2009 16 3437 3481 10.1245/s10434-009-0685-0 19777189

